# Shift work and quality of sleep: effect of working in designed dynamic light

**DOI:** 10.1007/s00420-015-1051-0

**Published:** 2015-04-19

**Authors:** Hanne Irene Jensen, Jakob Markvart, René Holst, Tina Damgaard Thomsen, Jette West Larsen, Dorthe Maria Eg, Lisa Seest Nielsen

**Affiliations:** Department of Anaesthesiology and Intensive Care, Kolding Hospital, Skovvangen 2-8, 6000 Kolding, Denmark; Energy and Environment, Danish Building Research Institute, Aalborg University, A.C. Meyers Vænge 15, 2450 København SV, Denmark; Institute of Regional Health Research, University of Southern Denmark, J.B. Winsløws Vej 19, 5000 Odense C, Denmark; Department of Anaesthesiology and Intensive Care, Vejle Hospital, Kabbeltoft 25, 7100 Vejle, Denmark

**Keywords:** Work environment, Shift work, Health, Sleep efficiency, Melatonin, Sleep monitoring

## Abstract

**Purpose:**

To examine the effect of designed dynamic light on staff’s quality of sleep with regard to sleep efficiency, level of melatonin in saliva, and subjective perceptions of quality of sleep.

**Methods:**

An intervention group working in designed dynamic light was compared with a control group working in ordinary institutional light at two comparable intensive care units (ICUs). The study included examining (1) melatonin profiles obtained from saliva samples, (2) quality of sleep in terms of sleep efficiency, number of awakenings and subjective assessment of sleep through the use of sleep monitors and sleep diaries, and (3) subjective perceptions of well-being, health, and sleep quality using a questionnaire. Light conditions were measured at both locations.

**Results:**

A total of 113 nurses (88 %) participated. There were no significant differences between the two groups regarding personal characteristics, and no significant differences in total sleep efficiency or melatonin level were found. The intervention group felt more rested (OR 2.03, *p* = 0.003) and assessed their condition on awakening as better than the control group (OR 2.35, *p* = 0.001). Intervention-ICU nurses received far more light both during day and evening shifts compared to the control-ICU.

**Conclusions:**

The study found no significant differences in monitored sleep efficiency and melatonin level. Nurses from the intervention-ICU subjectively assessed their sleep as more effective than participants from the control-ICU.

**Electronic supplementary material:**

The online version of this article (doi:10.1007/s00420-015-1051-0) contains supplementary material, which is available to authorized users.

## Introduction

An optimal circadian rhythm requires work and daily activities during daytime and sleep at night. However, a substantial number of work areas necessitate staff to work evenings and nights. About 20 % of the workforce in the Western world work fully or partially in shifts (Fritschi et al. 2011), with shift work being defined as work outside the timeframe of 6 a.m.–6 p.m. (Khosro et al. 2011). Hospital departments with acute functions, such as intensive care units (ICUs), have a higher percentage of shift workers than 20 %.

Persons working in shifts are often affected by fatigue (Akerstedt and Wright 2009), poor quality of sleep (Akerstedt et al. 2010; Axelsson et al. 2004), and metabolic disturbances (Wang et al. 2011). Furthermore, there is a plausible correlation between shift work and cardiovascular disease (Frost et al. 2009), different kind of cancers (Fritschi et al. 2011; Pesch et al. 2010; Wang et al. 2011), and other chronic diseases (Wang et al. 2011).

Thus, there is evidence correlating shift work with quality of sleep and chronic diseases, but a number of other factors such as lifestyle, work load, and physiological stress may also have an impact on quality of sleep and chronic disease (Khamisa et al. 2013).

Disturbance in melatonin production is one of the reasons for the found correlations between shift work and health issues (Fritschi et al. 2011; Haus and Smolensky 2006). The circadian rhythm, the sleep and wake cycle, is among other things influenced by the hormone melatonin (Borjigin et al. 2012). Melatonin production is suppressed by light and therefore normally highly suppressed during the day, increased during the evening and is high at nightfall (Mirick and Davis 2008). In evening or night shifts where work is conducted in conventional artificial light, the production of melatonin will be suppressed (Mirick and Davis 2008).

Another factor in connection with sleep disturbances is exposure to light during daytime where bright light exposure has been proved effective in minimising sleep disorders and fatigue (McCurry et al. 2011; Munch and Bromundt 2012). Light is considered the main human time keeper, and it is most likely the combination of light exposure both during the day and night that is important (Czeisler 2013; Lucas et al. 2014; Neil-Sztramko et al. 2014).

A recent review by Neil-Sztramko et al. (2014) showed heterogeneous results when looking at effects of controlled light exposure on health; some studies showed improved sleep efficiency, other studies not, and also in regard to correlation between bright light intervention and melatonin levels different results were found. Additional studies not included in the review also showed different results. Rahman et al. (2009) showed that by softening the evening light, it was possible to increase the melatonin level among persons with sleep difficulties due to low melatonin production, and Kayumov et al. (2005) showed that shift workers use of glasses that shielded for light entailed a normal increase in their production of melatonin. However, studies by Dumont et al. (2012) and Grundy et al. (2011) have failed to find the same effect. Dumont et al. (2012) found no evidence of direct melatonin suppression during night work among shift workers, but the study did suggest an association between higher light intensity during night work and internal circadian desynchrony. Grundy et al. (2011) found among 123 rotating shift nurses only a small relationship between change in melatonin level and light exposure during night shifts.

Further research in normal working conditions and workplaces is therefore needed to clarify the relation between light exposure, melatonin level and quality of sleep.

The purpose of the study was to examine the effect of designed dynamic light on quality of sleep with regard to sleep efficiency, level of melatonin in saliva, and subjective perceptions of quality of sleep.

## Materials and methods

### Design and hypotheses

Prospective intervention study where the effect of the designed dynamic light was examined. ICU staff working in designed dynamic light (intervention group) was compared with staff from a similar ICU with ordinary light (control group).

Primary outcomes were differences in sleep efficiency, level of melatonin, and subjective assessments of sleep quality. Secondary outcome was assessment of work environment.

The main clinical hypotheses were as follows: (1) sleep efficiency and the melatonin level at night would be higher for the intervention group compared to the control group and (2) the intervention group would subjectively assess their sleep as more effective compared to the control group.

### Designed dynamic light in intervention-ICU

In 2011, a Danish ICU was renovated. As part of the renovation, designed dynamic light that changes colour and intensity with time of the day and the work rhythm was installed. The aim was to create lighting conditions as close to daylight variations as possible. The lighting during the day imitated natural daylight, while the light in evening and night changed colour to prevent the suppression of melatonin production. Artificial daylight was made by using warm and cool white fluorescent light tubes with Ra >90, supplemented with tubes of the three primary colours red, green, and blue. Equally spaced 60 × 60 cm ceiling luminaires were Kelvin temperature-controlled using light tubes of the colours 827 and 965, while indirect lighting was provided by 4 × 54 W RGBW up lights, colour 60, 66, 67, and 965 to imitate the reflection of the sun on surroundings in patient rooms and the observation area. All luminaires were glare- and shadow-free with micro prismatic covers. The light changed colour and intensity with time of the day and the work rhythm. Between 5 a.m. and 6 a.m., the light setting gradually shifted from a nightlight to a daylight scenario, which was white in appearance and generally well lit. At 2 p.m., the light intensity decreased slightly to levels just exceeding the light requirements for hospitals (Danish Standard 703 1983). Between 8 p.m. and 10 p.m., a change occurred towards a mix of primarily red, green, and white light. The nightlight between 10 p.m. and 5 a.m. was dim, and the melatonin suppressing blue light was diminished causing the light to appear unnaturally red. In addition, it was in the patient rooms possible to overrule the general electrical lighting by selecting a number of specialised settings: acute, calming, IV catheter, ultra sound, and surveillance, thereby providing safe lighting conditions for all aspects of care. None of the personal computers were modified with diminished blue light during night shifts.

### Lighting in control-ICU

Warm fluorescent light from tubes of the colour 830 placed in equally spaced 60 × 60 cm ceiling luminaires provided the general lighting at the control-ICU with a Ra >85. The ceiling luminaires could be controlled by manual switches in three levels: off, 66 and 100 %. Unlike the corridors, daylight was available during daytime in the observation rooms and patient rooms from windows, but without direct sunlight due to tall buildings close by. The general lighting in the observation rooms was furthermore supplemented by movable desk lamps at each workstation. Movable task lamps and white fluorescent dimmable up lights were used in the patient rooms.

### ICU characteristics

The intervention-ICU was a secondary general ICU with 11 ICU beds, three intermediary care beds, and 14 recovery beds. The ICU received patients from medical and surgical specialities including trauma patients and cardiovascular surgery patients. In the intervention-ICU, the staff had their observation area in a central room with patient rooms all around. The central room was integrated with the corridors connecting to sluice rooms, drug room etc.

The control-ICU was likewise a secondary general ICU with eight ICU beds, one intermediary care bed and 14 recovery beds. Apart from the medical and surgical specialities, this ICU also received patients from the oncology speciality but no trauma patients. In the control-ICU, the staff was divided into two groups with one observation room each located between the patient rooms. The corridor linking the two groups and other rooms was separated from the observation rooms.

Daylight in the intervention-ICU was not present in the clean and unclean sluice room, negligible in the observation work areas, in the work desk areas, and corridors, but slightly present at the patient bedrooms. At the control-ICU, daylight was not present in the clean and unclean sluice room, and negligible in the corridors, but slightly present in the patient bedrooms, the observation rooms and the work desk areas.

At both ICUs, the whereabouts during work hours varied substantially from shift to shift, especially during night shifts, depending on number of patients and their severity of illness. With delirious or severely ill patients, much more time would be spent in the patient rooms for the individual staff member, whereas with stable sleeping or sedated patients, it would be a lot less. Table 1 shows an average estimation of distribution of time spent at different locations in the ICUs.

**Table 1:** Average estimation of the time staff spent in different locations in the ICU during different shifts

	Intervention-ICU			Control-ICU		
	Day (%)	Evening (%)	Night (%)	Day (%)	Evening (%)	Night (%)
Patient bedrooms	50	45	30	48	45	42
Observation room^a^	28	42	54	24	30	39
Corridor				9	9	3
Other^b^	22	13	16	19	16	16

^a^In the intervention-ICU, the observation room was integrated with the corridor

^b^Drug room, clean and unclean sluice rooms, coffee room, rest room

### Inclusion and exclusion criteria

Nursing staff (registered nurses and nursing assistants) working evening or night shifts, either full time or as part of their work rota, were included. Participants, normally working both evening and night shifts, participated in the project as belonging to either the evening or the night group, depending on which shifts they worked most. All staff who did not work in shifts (supervisors, management etc.), staff not having all of their work during shifts in the ICU (physicians), and staff not working in the designed dynamic light in their shifts were excluded.

### Participants

All nursing staff eligible for participating in the study received oral and written information about the study and was asked individually if they wanted to participate. Each participant was part of the study for ten consecutive days in which the participant each night slept with a sleep monitor and after each sleep period filled in a sleep diary. Furthermore, each participant provided saliva samples for a melatonin profile on either an evening shift (four samples) or night shift (five samples) and either on a day shift or a day off (two samples). Finally, during the 10-day period, all participants once filled in a general questionnaire about personal characteristics, experiences of quality of sleep both working shifts and not working shifts, and experienced effect of shift work on private life, health issues etc. Additionally, participants from the intervention-ICU filled in a questionnaire about use and experiences of the designed dynamic light.

In both ICUs, the shifts lasted 8 h and 15 min during weekdays and for some of the participants (equal in both ICUs) for 12 h during weekend. Due to the severity of illness of ICU patients, the number of staff at evening and night was close to the number of staff needed during daytime and therefore staff members had a median of 7–8 evening or night shifts a month. In both ICUs, staff overall planned their own work schedule based on individual wishes regarding number of shifts in a row etc., and in both ICUs day shifts began at 7 a.m., evening shifts at 3 pm, and night shifts at 11 p.m. except weekend 12-h shifts which began at 7 a.m. and 7 p.m.

### Data collection

Data collection was conducted from end of February 2013 until mid-May 2013. Participants were enrolled in the project due to availability of sleep monitors (ten available for each ICU) and to when they had a 10-day period with at least two evening or night shifts in a row, 2 dayshifts or 2 days off in a row, and with the possibility to return saliva samples collected at home within 48 h.

When enrolling in the study, each participant received an individual project package containing a sleep monitor (ready for use, coded with the individual’s project code number), a sleep diary with the appropriate dates already entered, information about how to collect saliva samples, a table showing which specific dates to collect saliva samples, and a kit for each saliva sample. All items were marked with the individual’s project code number. The project package was presented by either the project leader or one of the three nursing staff members of the project group. Each part of the project and each item of the package were orally described, using an average of 20 min per participants. The participants also had access to a mobile number they could call at all times if they had any questions during the data collection period. When enrolling in the study, the participants also received an e-mail with a link to the electronic questionnaire (questionnaires for participants from the intervention-ICU). When the participants finished their 10-day data collection period, the sleep monitor and the sleep diary were returned, data from the sleep monitor were downloaded, and the monitor was then coded for the next participant. Data collection quality was monitored throughout the data collection period.

### Sleep monitoring

All participants monitored their sleep during the 10-day period using Actisleep Monitors from ActiGraph. The sleep monitors recorded latency (time to sleep onset), number of awakenings, length of awakenings, and an overall sleep efficiency (measured in a percentage, where more than 85 % was considered a normal sleep). The recorded data were analysed using ActiLife software version 6.0. All participants received a copy of their individual sleep monitoring results.

### Sleep diary

During the sleep monitoring period, the participants filled in a sleep diary daily, recording when they went to sleep, when they got up, how long it took to fall asleep, and what kind of shift (or day off) they had before the sleep period in question. Furthermore, they daily rated the overall quality of their sleep (from level 1 to 5), the level of feeling rested (from level 1 to 5), and the level of feeling awake (from level 1 to 9) of each night’s sleep; each scale had 1 as the best level. They also recorded if they had slept at other times during the day and if there were specific reasons for a disturbed sleep (illness, noise, sick children etc.). The sleep diary was originally developed by the National Research Centre for the Working Environment (NRCWE), Copenhagen, which granted permission for the use in this project. The sleep diary was in Danish, but a non-validated English version is available as supplementary material.

### Melatonin samples

All participants gave saliva samples for melatonin analysis during (1) either an evening or a night shift and (2) either a day shift or a day off. Night shifts samples were collected at 9 p.m., at midnight, at 3 a.m., at 6 a.m. and before the participants went to sleep (or at the latest, 9 a.m.). For evening shifts, samples were collected at 9 pm, at midnight, at 3 am, and when the participants woke up. For day shift or day offs, samples were collected when the participants got up in the morning and just before they went to bed. The saliva samples were collected on the second shift in a row or the second day off and all samples consisted of between 1½ and 2 ml of saliva. The samples were immediately placed in a refrigerator either at the ICU or at home, and within 48 h transferred to the hospital laboratory where it was frozen to minus 80 °C. When data collection was completed, all samples were checked, sorted, and sent for analyses at the laboratory at Gelderse Vallei Hospital, the Netherlands.

### Questionnaires

During the 10-day period, the participants electronically filled in a general questionnaire with personal characteristics, experiences of quality of sleep both working shifts and not working shifts, and experienced effect of shift work on private life, health issues etc. The questionnaire was originally developed for night shift workers by NRCWE, which granted permission for its use in this project. Two versions were used; one for night shift workers and one adapted for evening shift workers. Additionally, a few questions were added regarding the participants’ general exposure to light and their perception of their work light. The adapted questionnaires were pilot-tested for face and content validity among a small group of nurses and based on their comments and a floor and ceiling effect a few changes were made, among other things an extension of response categories from three (originally: always, sometimes, never) to five (changed version: very often/always, often, sometimes, rarely, never). The updated questionnaires were then again pilot-tested.

Furthermore, the participants from the intervention group filled in a short questionnaire on their experiences of working in the designed dynamic light and how they used the different functions. This questionnaire was developed by The Danish Building Research Institute/Aalborg University, Energy and Environment, Copenhagen (SBi).

The questionnaires were in Danish, but non-validated English versions are available as supplementary material.

### Light measurements

Each of the two ICUs was divided into subareas, wherein measuring points in space were selected for doing both horizontal and vertical measurements of photopic illuminance (lux) using a Hagner E4-X Digital Luxmeter, Instr. No. 4111, with corresponding Hagner Detector (B. Hagner AB, Solna, Sweden). The subareas covered the area where the ICU staff worked during shifts and with more than two measuring spots/subareas per room. Comparable subareas and measuring points at the intervention-ICU and control-ICU, respectively, were selected and matched and further used for comparable analysis of the light environments. Comparable subareas at each ICUs included (1) two observation room areas, (2) two patient bedrooms, (3) the clean sluice room, (4) the unclean sluice room, (5) two working desks, and (6) the corridors. Light measurements were performed on a representative day (24 h) on strategically selected time points in accordance with the daily rhythm of light change at the two ICUs.

Horizontal and vertical lux measurements, referring to the orientation of the sensor, were taken 85 cm above the floor at the different selected subareas. Moreover, in the observation rooms, light was measured at the edge of two representative work desks horizontally at 85 cm and vertically at typical eye height of 120 cm above the floor when seated. These measurements can be considered to be a measure of the light on the work desk for office tasks (working light) and the light perceived by a person sitting at a work desk, respectively.

Actiwatch Spectrum™ devices were used to document the diurnal rhythm of the light change being mounted vertically on the walls at a height of 1.5 m and spaced to cover the areas. Further specification about the light measurements can be found as supplementary material.

### Pre-study

A pilot study with six participants (three from each ICU) was conducted in 2012, testing sleep monitoring, sleep diary, and collection, transportation and quality of saliva samples.

Twenty sleep monitors were used in the project, and before data collection, all monitors were tested in random intervals between 2 and 10 nights by the study group and their relatives; based on the monitored data, the data analysis software was also tested. Furthermore, all monitors were tested for concordance by being placed on a rotating device for 22 h. The mean registration of rotations was for two different axis, 587.960 (CI 579.386; 596.534) and 591.884 (CI 580.658; 603.111), respectively.

### Statistical analyses

Background characteristics and questionnaire: univariate comparisons between the control and the intervention groups were done by the Mann–Whitney *U* test, Chi-square test, or Fischer’s exact test where appropriate for a range of key statistics. Data were analysed using Stata version 13 (Juul 2006).

For data from sleep diary (ordered variables), sleep monitoring and melatonin samples, various regression types were used for addressing the questions posed by the clinical hypotheses and analyses were performed using R (R Development Core Team 2014).

#### Sleep diary

The ordered categorical response variables were analysed by use of proportional odds, using the ‘ordinal’ R-package. Sleep monitoring and melatonin analyses: the number of awakenings were analysed by a Poisson model, whereas sleep efficiency and level of melatonin were analysed by normal regression models after logit and log transformations, respectively. For these analyses, the ‘lme4’ R-package was used.

The correlations induced by multiple measurements were addressed by using individuals as a random effect. The indicator for control or intervention group was used as a primary exposure variable. Other covariates included age, body mass index (BMI), duration of sleep, shift work, and actual shift (sleep after day off or a day, evening or night shift). Suitable sine and cosine terms were included to account for measurements being taken at different times of the year and at different time of the day (melatonin). The models were reduced, by removing insignificant effects using likelihood-ratio tests. Some measurements of melatonin could only be determined to be below 0.5 pg/ml or above 50 pg/ml. These are thus partly missing data and were therefore handled by multiple imputations with suitable constraints on the sampling domain.

### Ethics

The project was approved by The Regional Scientific Ethical Committees for Southern Denmark (S-20110140) and was registered with the Danish Data Protection Agency. Informed consent was obtained from all individual participants included in the study. All procedures performed in the study were in accordance with the ethical standards of the Regional Scientific Ethical Committees and with the 1964 Helsinki declaration and its later amendments.

## Results

A total of 55 nurses (89 %) from the intervention-ICU and 58 nurses (88 %) from the control-ICU participated in the project.

As shown in Table 2, the only significant difference between the two groups was regarding shift work; both in number of shifts per months and in shift combinations.

**Table 2:** Background characteristics of participants

	Intervention-ICU (*n* = 55)		Control-ICU (*n* = 58)		*p* ^b^
	Median	Range^a^	Median	Range^a^	
Age	43	(35–50)	42	(36–53)	0.74
Years employed in the ICU	10	(4–20)	10	(5–21)	0.40
Work hours per week	37	(33–37)	34	(32–37)	0.44
Number of shifts per month^c^	8	(6–10)	7	(5–8)	0.02

	*N*	%	*N*	%	*p* ^d^
Live with partner	46	(84)	47	(81)	0.72
Children living at home	36	(65)	35	(60)	0.57
Shift type					<0.001
Day/evening	11	(20)	23	(40)	
Day/evening/night	10	(18)	22	(39)	
Day/night	27	(49)	11	(19)	
Only evening	5	(9)	0		
Only night	2	(4)	1	(2)	
Years with shift work					0.69
<1 year	1	(2)	1	(2)	
1 to <10 years	26	(47)	23	(40)	
10 to <20 years	18	(33)	18	(31)	
20 years or more	10	(18)	16	(28)	

^a^Interquartile range

^b^Mann–Whitney *U* test

^c^Excluding those working full time evening or night

^d^Chi-square test or Fischer’s exact test where appropriate

Due to the differences in shift combinations, the intervention group participated with 40 % in the overall evening shift group and 60 % in the night shift group, whereas the control group participated with 60 % in the evening shift group and 40 % in the night shift group (*p* = 0.03).

No significant differences were found between the intervention group and the control group for a number of other areas asked, such as time of transport to work, influence of shift work on family life, and spare time activities, assessment of general physical health, hours spent outdoors, level of light in their bed room etc. (results not shown).

### Sleep monitoring

No significant differences in sleep efficiency or length of awakenings were found between the two groups, but the nurses in the control-ICU had 16 % more awakenings (*p* = 0.05). Adjusted analyses showed that for a participant aged 43, BMI 24, and at day 83 of the year, the mean sleep efficiency after day shifts was 89.5 % (CI 88.3; 90.7 %), after evening shift was 87.5 % (CI 86.5; 89.3 %), after night shift was 90.9 % (CI 89.8; 91.9 %), and after days off was 88.6 % (CI 87.4; 89.8 %). Sleep efficiency above 85 % was defined as normal sleep.

Monitored time to onset of sleep was not in accordance with self-reported time to onset.

### Sleep diary

Table 3 shows the participants’ own assessment of their sleep during the 10-day data collection period, controlled/adjusted for possible confounders (as described in the “Statistical analyses” section).

**Table 3:** Sleep diary

	Quality of sleep period^a^			Degree of feeling rested^a^			Condition at awakening^a^		
	OR	CI	*p* ^b^	OR	CI	*p* ^b^	OR	CI	*p* ^b^
ICU^c^	–	– –	–	2.03	(1.28; 3.22)	0.003	2.35	(1.40; 3.94)	0.001
Shift group^d^	0.57	(0.36; 0.88)	0.01	–	– –	–	–	– –	–
Actual shift: evening^e^	0.80	(0.42; 1.52)	0.49	1.81	(1.18; 2.77)	0.006	1.73	(1.13; 2.65)	0.01
Actual shift: night^e^	0.52	(0.28; 0.96)	0.04	0.95	(0.61; 1.49)	0.84	0.93	(0.60; 1.46)	0.76
Actual shift: day off^e^	0.56	(0.33; 0.95)	0.03	0.89	(0.63; 1.25)	0.50	0.78	(0.56; 1.10)	0.16
Day shift (ICU)^f^	0.71	(0.36; 1.41)	0.32	–	– –	–	–	– –	–
Evening shift × ICU^f^	1.51	(0.77; 2.95)	0.23	–	– –	–	–	– –	–
Night shift × ICU^f^	2.22	(1.10; 4.46)	0.03	–	– –	–	–	– –	–
Day off × ICU^f^	1.39	(0.85; 2.28)	0.19	–	– –	–	–	– –	–
Duration of sleep	0.97	(0.88; 1.07)	0.53	0.74	(0.67; 0.82)	<0.001	0.78	(0.71; 0.86)	<0.001
BMI	0.99	(0.95; 1.04)	0.78	0.99	(0.94; 1.04)	0.56	0.98	(0.93; 1.03)	0.35
Age	0.99	(0.97; 1.01)	0.32	0.98	(0.96; 1.00)	0.12	0.95	(0.92; 0.98)	<0.001
Seasonal variation 1^g^	0.31	(0.02; 4.96)	0.41	0.07	(0.00; 1.26)	0.07	0.02	(0.00; 0.11)	<0.001
Seasonal variation 2^h^	1.95	(1.07; 3.57)	0.03	1.07	(0.54; 2.13)	0.84	1.75	(0.85; 3.61)	0.13

Daily assessment of sleep

^a^Quality of sleep/degree of feeling rested (scored from 1 to 5), condition at awakening (scored from 1 to 9); each scale had 1 as the best level

^b^Ordinal logistic regression

^c^Control-ICU versus intervention-ICU. “–” Variable did not contribute significantly to the association

^d^Night shift group versus evening shift group

^e^Actual shift (evening, night and day off) versus day shift

^f^Interaction between ICU and shift

^g^Sine variation

^h^Cosine variation

For assessment of the quality of sleep, no general difference was found between the two ICUs; but after night shifts, the intervention group assessed their sleep as having higher quality than the control group (OR 2.22, *p* = 0.03). The intervention group felt more rested (OR 2.03, *p* = 0.003) and assessed their condition on awakening as better than the control group (OR 2.35, *p* = 0.001).

### Melatonin

**Fig. 1:**
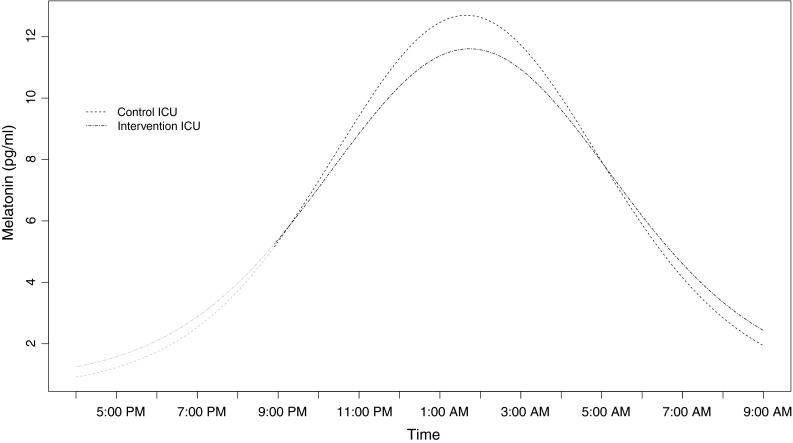
Average melatonin profiles for intervention-ICU and control-ICU. Based on all melatonin samples from an evening or night shift [9 p.m., midnight, 3 a.m., 6 a.m. (only night shifts), and awakening or before bed] and at awakening/before sleep on day shift or day off. The results were adjusted for shift, BMI, age and time of year

Table 4 presents an overview of unadjusted melatonin levels at different time points (Table 4). When adjusted for possible confounders, no overall significant differences were found between intervention and control group regarding melatonin level (Fig. 1). Statistical model is available as supplementary material.

**Table 4:** Melatonin levels at different time points

	Intervention-ICU			Control-ICU			*p* ^b^
	*n*	Median	IQR^a^	*n*	Median	IQR^a^	
Evening shift group							
Evening shift 9 p.m.	22	1.8	(0.6; 2.4)	33	2.6	(0.8; 8.1)	0.05
Evening shift midnight	22	7.0	(4.6; 14.7)	34	10.5	(7.6; 27.8)	0.14
Evening shift 3 a.m.	20	10.5	(3.7; 50.0)	33	15.9	(9.3; 41.2)	0.25
Evening shift at awakening (between 6 and 9)	20	4.8	(3.6; 7.1)	34	4.3	(1.7; 6.3)	0.13
Day shift. At time of awakening	6	9.4	(6.3; 25.5)	17	7.2	(4.5; 14.2)	0.53
Day shift. Before sleep at night	6	10.7	(8.8; 16.2)	18	7.2	(2.9; 10.2)	0.05
Day off. At time of awakening	16	4.8	(3.6; 12.8)	16	4.7	(2.9; 6.8)	0.58
Day off. Before sleep at night	15	14.8	(4.6; 47.4)	16	6.5	(3.2; 18.9)	0.22
Night shift group							
Night shift 9 p.m.	32	3.2	(1.3; 6.7)	21	1.1	(0.5; 2.6)	0.05
Night shift midnight	32	10.3	(3.6; 15.5)	22	10.2	(2.7; 21.3)	0.89
Night shift 3 a.m.	32	12.7	(5.3; 17.7)	23	15.0	(2.5; 24.3)	0.77
Night shift 6 a.m.	31	7.1	(3.8; 19.7)	23	8.0	(4.2; 18.7)	0.75
Night shift before sleep (between 8 and 9 a.m.)	32	2.0	(0.9; 4.2)	22	2.4	(1.0; 5.3)	0.46
Day shift. At time of awakening	10	8.4	(3.9; 18.3)	13	8.7	(4.7; 33.5)	0.51
Day shift. Before sleep at night	10	8.8	(6.1; 14.3)	13	6.6	(3.0; 17.0)	0.51
Day off. At time of awakening	22	4.7	(1.6; 12.9)	7	3.9	(1.8; 14.6)	0.84
Day off. Before sleep at night	23	12.2	(3.8; 16.5)	8	8.0	(1.9; 10.6)	0.05

Comparison between intervention-ICU and control-ICU

^a^Interquartile range

^b^Mann–Whitney *U* test

For both groups, there were considerable individual variations with a minimum melatonin level range from <0.5 to 3.7 pg/ml and a maximum level range from 4.8 to >50 pg/ml.

### Questionnaires

When comparing the participants’ general perceptions of quality of sleep, the intervention group found it easier to fall asleep after shift work and felt more rested after sleep both after shifts or non-shifts compared to the control group (Table 5).

**Table 5:** Participants’ general experiences of sleep

	Very often/always		Often		Sometimes		Rarely		Never		*p* ^a^
	*n*	%	*n*	%	*n*	%	*n*	%	*n*	%	
Difficulties falling asleep when not working shifts											0.39
Intervention-ICU	1	(1.8)	2	(3.6)	23	(41.8)	26	(47.3)	3	(5.5)	
Control-ICU	4	(7.0)	7	(12.3)	16	(28.1)	29	(50.9)	1	(1.8)	
Difficulties falling asleep after shift work											0.01
Intervention-ICU	3	(5.6)	2	(3.7)	10	(18.5)	25	(46.3)	14	(25.9)	
Control-ICU	8	(13.8)	1	(1.7)	18	(31.0)	25	(43.1)	6	(10.3)	
Restless and interrupted sleep when not working shifts											0.09
Intervention-ICU	4	(7.3)	8	(14.6)	29	(53.7)	13	(23.6)	1	(1.8)	
Control-ICU	2	(3.5)	21	(36.2)	25	(43.1)	9	(15.5)	1	(1.7)	
Restless and interrupted sleep when working shifts											0.23
Intervention-ICU	4	(7.3)	11	(20.0)	24	(43.6)	14	(25.5)	2	(3.6)	
Control-ICU	3	(5.2)	10	(17.2)	20	(34.5)	25	(43.1)	0	(0.0)	
Rested after sleep when not working shifts											0.02
Intervention-ICU	9	(16.4)	33	(60.0)	11	(20.0)	2	(3.6)	–	–	
Control-ICU	3	(5.2)	32	(55.2)	19	(32.8)	4	(6.9)	–	–	
Rested after sleep (evening shifts)											0.04
Intervention-ICU	4	(19.1)	10	(47.6)	3	(14.3)	4	(19.1)	–	–	
Control-ICU	2	(5.7)	10	(28.6)	14	(40.0)	8	(22.9)	1	(2.9)	

^a^Mann–Whitney *U* test

When asked to choose three words that best characterised their work light (out of ten options), the three main words for the intervention group were pleasant (84 vs. control 14 %), relaxing (65 vs. control 5 %), and natural (42 vs. control 7 %), whereas the three main words for the control group were institutional (84 vs. intervention 20 %), artificial (74 vs. intervention 35 %), and gloomy (40 vs. intervention 2 %).

### Light measurements

Both ICUs were characterised as having none or limited daylight availability. Measurements at selected and comparable spots showed generally higher lux levels at the intervention-ICU in comparison with the control-ICU (Table 6).

**Table 6:** Horizontally and vertically measured lux at centre of two observation rooms, the clean and unclean sluice room; intervention and control-ICU, respectively (*n* = 2)

	Average vertical lux		Average horizontal lux	
	Intervention-ICU	Control-ICU	Intervention-ICU	Control-ICU
Observation room 1	386	192^a^	590	434^a^
Observation room 2	346	73^a^	451	69^a^
Patient bed room 1	519^a^	195^a^	548^a^	440^a^
Patient bed room 2	674^a^	80^a^	683^a^	98^a^
Clean sluice room	378	173	906	397
Unclean sluice room	378	100	906	430
Work desk daytime^b^	409	230	639	496
Work desk night-time	114^d^	150^c^	142^d^	300^c^

^a^Data influenced by limited daylight availability

^b^Vertical lux measured at eye level 120 cm above ground, unlike the above listed locations where the vertical lux was measure at 85 cm above ground

^c^Great variations depending on the position of the movable desk lamp

^d^The light was red in appearance

The average perceived light measured at work desks was significantly higher at the intervention-ICU compared to the control-ICU during the day, i.e. approximately 29 % horizontally measured (vertically distributed) and 78 % vertically measured (horizontally distributed) higher illuminance (Table 6). During the nights, more light was present at the control-ICU work desks where the estimated time spent (at the observation rooms) was up 39 %. Moreover, R:G:B contribution (%) with dominating red light in the intervention-ICU indicated greater melatonin suppressing effect in the control-ICU compared to the intervention-ICU.

The estimated time spent in the intervention-ICU observation room integrated with corridor was during the day 28 %, evening 42 % and night 54 %. The light at the intervention-ICU corridors equalled more or less the light at the work areas, resulting in average horizontally measured lux/vertically measured lux in the morning (9 a.m.) = 525/292 lux, in the afternoon (3 p.m.) = 579/362 lux, and evening (7.15 p.m.) = 433/246 lux, which in average = 513/300 lux. This results in the horizontally/vertically measured lux relation of 1.71. During the night (11:45 p.m.), this was measured to be 1:15 (68/59 lux); thus, there were more vertically measured lux during the night than during the day in relation to the horizontally measured lux 85 cm above the floor.

At 100 % intensity, the general lighting at the control-ICU corridor was 311/89 lux = 3.49 and at 66 % intensity 209/60 lux = 3.48. At the control-ICU corridor where the staff spent <10 % of their time, there were much less vertically measured lux during the day and similar vertically measured lux during the night as for the intervention-ICU corridor, but the spectral compositions of light during the night were significantly different.

The light at the control-ICU was in general more vertically and downward distributed compared to the light at the intervention-ICU. Therefore, nurses working in the control-ICU received less light at the cornea compared with nurses working in the intervention-ICU, in particular during daytime.

**Fig. 2:**
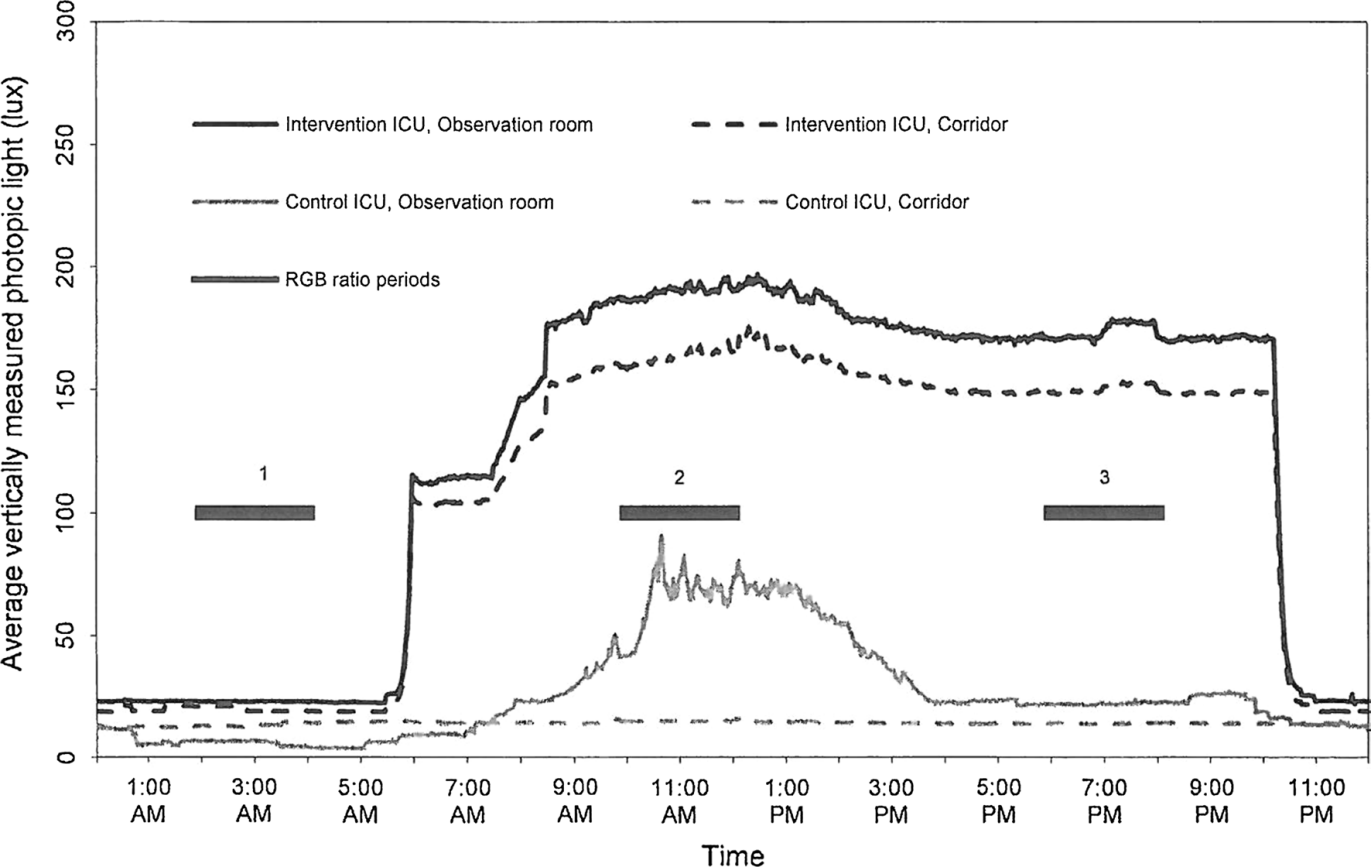
Light measurements. Measurements were logged per minute at 150 cm above the floor level at the two ICUs during 1 week (i.e. every minute point contains an average of data from 7 days). The *dashed lines* representing the corridors are measurements of ten subareas (*n* = 10) and the *solid lines* represents the observation room with measurements of two subareas (*n* = 2)

The diurnal light rhythm documented with the Actiwatches showed that light in the control-ICU observation room and corridors seemed to be turned on more or less at all times including the nights (Fig. 2). During daytime, the dominating measured Actiwatch colour was green for the intervention-ICU, whereas red and green was both relatively high for the control-ICU (Table 5). During the nights, the red light clearly dominated the nightlight at the intervention-ICU, whereas nightlight at the control-ICU was not found to change significantly between day and night (Fig. 2; Table 7).

**Table 7:** The ratio of Actiwatch R, G and B light^a^ of the periods: (1) between 2 a.m. and 4 a.m., (2) between 10 a.m. and noon, and (3) between 6 p.m. and 8 p.m.

RGB ratio periods (see Fig. 2)	Intervention-ICU, observation room R:G:B contribution (%)	Control-ICU, observation room R:G:B contribution (%)	Intervention-ICU, corridor R:G:B contribution (%)	Control-ICU, corridor R:G:B contribution (%)
1	76:17:7	56:40:4	76:17:7	54:41:6
2	24:56:21	42:46:12	26:55:19	54:40:6
3	23:56:21	57:39:4	26:55:19	54:40:6

^a^Spectra of light associated with the colour perceptions of red (R light), green (G light) and blue (B light), respectively

Light intensity differences between the ICUs were found during the daytime, where low lux levels were measured in the control-ICU observation rooms, patient bedrooms, corridors, and other selected rooms. Therefore, the intervention-ICU staff received more light both during the day and evening than the control-ICU staff, when at work. During the nights, estimated time spent at the control-ICU observation room area was up to 39 %. Movable desk lamps with white light were used at the control-ICU, while the main night-time light at the intervention-ICU was red.

## Discussion

How to minimise the negative effects of shift work is an ongoing debate and improving the work environment is one of many options being investigated. In this study, the effects of a dynamic regulated lighting scheme were studied. No significant differences in monitored sleep efficiency and melatonin level were found. Nurses from the intervention-ICU found the dynamic light agreeable and subjectively assessed their sleep as more effective than participants from the control-ICU.

The light measurements showed that the intervention-ICU staff received far more light both during the day and evening than the control-ICU staff when at work. During the night, the light differences in melatonin suppressing light were mainly due to colour differences being red in the intervention-ICU and a mix of red and green in the control-ICU. The red light hardly suppresses the melatonin production which is mostly influenced by the blue part of the spectrum (Thapan et al. 2001; Brainard et al. 2001). During night-time, the blue light contribution was low in both ICUs, but the green light may also have caused melatonin suppression in the control-ICU at night. However, the diurnal light variation affecting the circadian rhythm was small at the control-ICU compared to the variation at the intervention-ICU and this may have influenced the results found on sleep quality. The main reasoning is that bright light exposure during the day of certain duration may be effective in enhancing the coherence of the sleep–wake cycle (Shikder et al. 2012; Sloane et al. 2008). Besides, the spatial light distribution clearly differed between the ICUs resulting in a more even horizontal versus vertical light distribution at the intervention-ICU. This most likely contributed to the perception of the work light being agreeable for the intervention group and gloomy for the control group.

The substantial differences found in individual melatonin levels are in accordance with other measurements (Burgess and Fogg 2008) and may partly explains the lack of significant differences between the melatonin levels of the ICU groups. Melatonin was measured in saliva which is a non-invasive, practical and often used method for melatonin analyses (Mirick and Davis 2008). One of the issues when using saliva samples is to get sufficient saliva for analyses; in this study, only 18 out of 734 samples (2.5 %) were insufficient for analysis. For practical reasons, the shift profiles only consisted of four samples for evening shift workers and five samples for night shift workers. With substantial individual variations, the actual peak levels may not have been measured (Burgess and Fogg 2008).

### Strengths of the study

The high participation rate minimised selection biases, and the multi-method approach provided a nuanced description of staff perceptions of quality of sleep. The study was delayed 2½ years after the designed dynamic light was installed which minimizes the effect of experiences of improvements just due to changes. All sleep monitors were pre-tested both individually and for concordance measuring.

### Limitations of the study

As there were wide individual differences in sleep patterns and melatonin level, a paired before–after design would have reduced the risk of confounders considerably. When the intervention-ICU was renovated, designed dynamic light was not originally planned but was incorporated during the process, and therefore, it was not possible to plan and conduct an intervention study in a paired before–after design. Although the control group was from the same region, the ICUs had similar ways of working (for example a high nurse–patient ratio due to the severity of the patients’ conditions which meant that in most of their shift, the nurses were in or close to the patient rooms), and no differences were found for personal characteristics, there were differences in distribution of shifts, in layout of the ICUs, and possibly also in other, unknown factors between the ICUs. The estimated average whereabouts during work hours varied between the ICUs, and whereabouts also varied substantially from shift to shift in both ICUs. This may have blurred a perhaps existing effect of designed dynamic light on melatonin level and sleep efficiency. If the control-ICU at some point installs designed dynamic light, it will be possible to conduct a before–after study with results from this study as baseline data.

Melatonin level can change rapidly with altered light conditions (Wahnschaffe et al. 2013). The time points for measurements were decided both based on the literature (Kayumov et al. 2005) and in concordance with a study planned by the Danish National Research Centre for the Working Environment in order to be able to compare results. The second time point in this study was midnight, which was relevant for night shift workers who began their shift at 11 p.m., but for evening shift workers, 11 p.m. would probably have been a better measurement time point as they got off duty between 11:15 p.m. and midnight and as such may have been exposed to outside work lighting before their midnight sample. However, this was the same for evening shift workers in both the intervention and control group. The selected time points therefore did not influence the difference between the groups, but may have had an effect on the general melatonin level.

The recent review by Neil-Sztramko et al. (2014) examining health-related interventions among night shift workers found that a combination of timed bright light and light-blocking goggles may promote adaption to shift work, but heterogeneous results were found among the included studies. One of the reasons for not finding differences between the two groups in sleep efficiency and level of melatonin in this study may be the exposure to light outside the ICUs. Intervention studies in normal working conditions and without the possibility to randomise or blind the participants involve risks of confounders and biases which may influence the chance to prove an effect of the intervention.

There was high agreement between monitored sleep and self-reported sleeping time and quality of sleep (sleep diary), but not for time to onset of sleep. The Actisleep monitors registered onset of sleep if the participants were very still even though awake. Therefore, monitored time to onset of sleep was not analysed. However, time to onset of sleep was included in total sleep efficiency indirectly, as extra numbers of awakenings were counted when participants turned around while trying to fall asleep.

The participants were asked about the number of hours they usually spent outdoors and level of light in their bedroom, and no significant differences were found between the two groups. If the participants had worn a light logger, it would have been possible to compare both the precise whereabouts in the ICUs and validate the self-reported light conditions of outside work environments.

The differences found between the intervention group and the control group regarding how difficult it was to fall asleep after shift work and the degree of feeling rested after sleep after shifts or non-shifts may be influenced by potential confounders outside the ICUs. The tendency to unequal distribution in numbers of years working shift schedules between the two groups indicates a higher adaption to shift work for the control group. This could bias the results in two ways: either strengthen the results if adaption means better sleep or weaken the results if sleep after many years of shift work becomes more difficult.

While no significant effect of designed dynamic light on the ICU nurses’ objective measured sleep quality was found, the study showed that lighting influenced the subjective well-being of staff working shifts. The differences in illuminances for the two ICUs in daytime may be a contributory cause. The differences in the diurnal light fluctuation possibly maintained the nurses’ circadian rhythm better in the intervention-ICU than in the control-ICU. When rebuilding and planning new institutions, lighting is an important part in order to create a work environment which is as agreeable as possible for workers, and which contributes to minimising the negative effects of shift work (Brawley 2009).

In conclusion, the study found no significant differences in monitored sleep efficiency and melatonin level. Nurses from the intervention-ICU found the dynamic light agreeable and subjectively assessed their sleep as more effective than participants from the control-ICU. The study indicates that designed dynamic light may influence quality of sleep positively.

## Electronic supplementary material

Supplementary material 1 (XML 183 kb)

Supplementary material 2 (DOC 58 kb)

Supplementary material 3 (DOC 24 kb)

Supplementary material 4 (DOC 24 kb)

## References

[CR2] Akerstedt T, Wright KP (2009). Sleep loss and fatigue in shift work and shift work disorder. Sleep Med Clin.

[CR3] Akerstedt T, Nordin M, Alfredsson L, Westerholm P, Kecklund G (2010). Sleep and sleepiness: impact of entering or leaving shiftwork—a prospective study. Chronobiol Int.

[CR4] Axelsson J, Akerstedt T, Kecklund G, Lowden A (2004). Tolerance to shift work-how does it relate to sleep and wakefulness?. Int Arch Occup Environ Health.

[CR5] Borjigin J, Zhang LS, Calinescu AA (2012). Circadian regulation of pineal gland rhythmicity. Mol Cell Endocrinol.

[CR6] Brainard GC, Hanifin JP, Greeson JM, Byrne B, Glickman G, Gerner E, Rollag MD (2001). Action spectrum for melatonin regulation in humans: evidence for a novel circadian photoreceptor. J Neurosci.

[CR7] Brawley EC (2009). Enriching lighting design. NeuroRehabilitation.

[CR8] Burgess HJ, Fogg LF (2008). Individual differences in the amount and timing of salivary melatonin secretion. PLoS One.

[CR9] Czeisler CA (2013). Casting light on sleep deficiency. Nature.

[CR10] Danish Standard (DS) 703 (1983) Guidelines for artificial illumination in hospitals, 3rd edn. Danish Council of Standardisation, Copenhagen, pp 1–10 (in Danish)

[CR11] Dumont M, Lanctot V, Cadieux-Viau R, Paquet J (2012). Melatonin production and light exposure of rotating night workers. Chronobiol Int.

[CR12] Fritschi L, Glass DC, Heyworth JS, Aronson K, Girschik J, Boyle T, Grundy A, Erren TC (2011). Hypotheses for mechanisms linking shiftwork and cancer. Med Hypotheses.

[CR13] Frost P, Kolstad HA, Bonde JP (2009). Shift work and the risk of ischemic heart disease—a systematic review of the epidemiologic evidence. Scand J Work Environ Health.

[CR14] Grundy A, Tranmer J, Richardson H, Graham CH, Aronson KJ (2011). The influence of light at night exposure on melatonin levels among Canadian rotating shift nurses. Cancer Epidemiol Biomark Prev.

[CR15] Haus E, Smolensky M (2006). Biological clocks and shift work: circadian dysregulation and potential long-term effects. Cancer Causes Control.

[CR16] Juul S (2006). An introduction to Stata for health researchers.

[CR17] Kayumov L, Casper RF, Hawa RJ, Perelman B, Chung SA, Sokalsky S, Shapiro CM (2005). Blocking low-wavelength light prevents nocturnal melatonin suppression with no adverse effect on performance during simulated shift work. J Clin Endocrinol Metab.

[CR18] Khamisa N, Peltzer K, Oldenburg B (2013). Burnout in relation to specific contributing factors and health outcomes among nurses: a systematic review. Int J Environ Res Public Health.

[CR19] Khosro S, Alireza S, Omid A, Forough S (2011). Night work and inflammatory markers. Indian J Occup Environ Med.

[CR20] Lucas RJ, Peirson SN, Berson DM, Brown TM, Cooper HM, Czeisler CA (2014). Measuring and using light in the melanopsin age. Trends Neurosci.

[CR21] McCurry SM, Pike KC, Vitiello MV, Logsdon RG, Larson EB, Teri L (2011). Increasing walking and bright light exposure to improve sleep in community-dwelling persons with Alzheimer’s disease: results of a randomized, controlled trial. J Am Geriatr Soc.

[CR22] Mirick DK, Davis S (2008) Melatonin as a biomarker of circadian dysregulation. Cancer Epidemiol Biomark Prev 17:3306–3313 10.1158/1055-9965.EPI-08-0605 19064543

[CR23] Munch M, Bromundt V (2012). Light and chronobiology: implications for health and disease. Dialogues Clin Neurosci.

[CR24] Neil-Sztramko SE, Pahwa M, Deemers PA, Gotay CC (2014). Health-related interventions among shift workers: a critical review of the literature. Scand J Work Environ Health.

[CR25] Pesch B, Harth V, Rabstein S, Baisch C, Schiffermann M, Pallapies D (2010). Night work and breast cancer—results from the German GENICA study. Scand J Work Environ Health.

[CR26] R Development Core Team (2014). R: a language and environment for statistical computing.

[CR27] Rahman SA, Kayumov L, Tchmoutina EA, Shapiro CM (2009). Clinical efficacy of dim light melatonin onset testing in diagnosing delayed sleep phase syndrome. Sleep Med.

[CR28] Shikder S, Mourshed M, Price A (2012). Therapeutic lighting design for the elderly: a review. Perspect Public Health.

[CR29] Sloane PD, Figueiro M, Cohen L (2008). Light as therapy for sleep disorders and depression in older adults. Clin Geriatr.

[CR30] Thapan K, Arendt J, Skene DJ (2001). An action spectrum for melatonin suppression: evidence for a novel non-rod, non-cone photoreceptor system in humans. J Physiol.

[CR31] Wahnschaffe A, Haedel S, Rodenbeck A, Stoll C, Rudolph H, Kozakov R, Schoepp H, Kunz D (2013). Out of the lab and into the bathroom: evening short-term exposure to conventional light suppresses melatonin and increases alertness perception. Int J Mol Sci.

[CR32] Wang XS, Armstrong ME, Cairns BJ, Key TJ, Travis RC (2011). Shift work and chronic disease: the epidemiological evidence. Occup Med (Lond).

