# Supplementary material for: Shift work and quality of sleep: effect of working in designed dynamic light
Source: Int Arch Occup Environ Health. 2015 Apr 19;89:49–61. doi: 10.1007/s00420-015-1051-0 (PMC4700071; doi:10.1007/s00420-015-1051-0)
Supplement: Supplementary file 2 — Supplementary material 2 (DOC 58 kb) [file 420_2015_1051_MOESM2_ESM.doc]

**Sleep diary** (to be filled in when you wake up) Project-id: ___________ Sleep monitor number ______________  **(identical sheet for day 6-10)**

|  | **Day 1** | **Day 2** | **Day 3** | **Day 4** | **Day 5** |
| --- | --- | --- | --- | --- | --- |
| **Date** for filling in the entry (e.g. 1304 or 13/4) |  |  |  |  |  |
| **Time** for filling in the entry, what time is it now? (e.g. 6:45) |  |  |  |  |  |
| **When did you go to sleep?** (switched off the light, e.g.22:06) |  |  |  |  |  |
| **How many minutes did it take to fall a sleep?** |  |  |  |  |  |
| **What was the time when you woke up?** (e.g. 6:05) |  |  |  |  |  |
| **What was the time when you got up?** (e.g. 6:13) |  |  |  |  |  |
| **How many times were you awake during the night?** |  |  |  |  |  |
| **How was your sleep?** 1 Very good  (mark just one number each day) 2 Quite good  3 Neither / nor  4 Quite bad  5 Very bad | 1 🞏  2 🞏  3 🞏  4 🞏  5 🞏 | 1 🞏  2 🞏  3 🞏  4 🞏  5 🞏 | 1 🞏  2 🞏  3 🞏  4 🞏  5 🞏 | 1 🞏  2 🞏  3 🞏  4 🞏  5 🞏 | 1 🞏  2 🞏  3 🞏  4 🞏  5 🞏 |
| **How rested did you feel?** 1 Totally  (mark just one number each day) 2  3 Some  4  5 Not at all | 1 🞏  2 🞏  3 🞏  4 🞏  5 🞏 | 1 🞏  2 🞏  3 🞏  4 🞏  5 🞏 | 1 🞏  2 🞏  3 🞏  4 🞏  5 🞏 | 1 🞏  2 🞏  3 🞏  4 🞏  5 🞏 | 1 🞏  2 🞏  3 🞏  4 🞏  5 🞏 |
| **How did you feel when you woke up?**  (mark just one number each day)  1 Very awake  2  3 Awake  4  5 Neither awake nor sleepy  6  7 Sleepy but no problem keeping awake  8  9 very sleepy, problem keeping awake, fighting with sleep | 1 🞏  2 🞏  3 🞏  4 🞏  5 🞏  6 🞏  7 🞏  8 🞏  9 🞏 | 1 🞏  2 🞏  3 🞏  4 🞏  5 🞏  6 🞏  7 🞏  8 🞏  9 🞏 | 1 🞏  2 🞏  3 🞏  4 🞏  5 🞏  6 🞏  7 🞏  8 🞏  9 🞏 | 1 🞏  2 🞏  3 🞏  4 🞏  5 🞏  6 🞏  7 🞏  8 🞏  9 🞏 | 1 🞏  2 🞏  3 🞏  4 🞏  5 🞏  6 🞏  7 🞏  8 🞏  9 🞏 |
| **What time did you begin work yesterday?**  (Please write DO, if you were not at work) |  |  |  |  |  |
| **Hvornår fik du fri i går?**  (Skriv F, hvis du ikke var på arbejde) |  |  |  |  |  |

**Please see next page**

**Please note, if something specific disturbed your sleep. For example**

- Drank too much coffee/tea or alcohol yesterday
- Noise, partner’s snoring
- Children
- Going to the lavatory
- Illness, e.g. coughing, infectious desease or pain
- Was thinking about problems at work or in the family

**Please also note if you have taken sleep medication or if you forgot to wear the sleep monitor.**

Day 1: ___________________________________________________________________________________

_________________________________________________________________________________________

_________________________________________________________________________________________

Day 2: ___________________________________________________________________________________

_________________________________________________________________________________________

_________________________________________________________________________________________

Day 3: ___________________________________________________________________________________

_________________________________________________________________________________________

_________________________________________________________________________________________

Day 4: ___________________________________________________________________________________

_________________________________________________________________________________________

_________________________________________________________________________________________

Day 5: ___________________________________________________________________________________

_________________________________________________________________________________________

_________________________________________________________________________________________
