# Supplementary material for: Shift work and quality of sleep: effect of working in designed dynamic light
Source: Int Arch Occup Environ Health. 2015 Apr 19;89:49–61. doi: 10.1007/s00420-015-1051-0 (PMC4700071; doi:10.1007/s00420-015-1051-0)
Supplement: Supplementary file 3 — Supplementary material 3 (DOC 24 kb) [file 420_2015_1051_MOESM3_ESM.doc]

**Supplementary material**

**Specification. Light measurements**

Lux measurements at the subareas were performed using a Hagner E4-X Digital Luxmeter, Instr. No. 4111, with corresponding Hagner Detector (B. Hagner AB, Solna, Sweeden). For the spectral light measurements a cosine corrected detector (CC-3-UV-S, Ocean Optics, Inc, FL 34698, USA) was used, which was connected via an optic fiber to an Ocean Optics HR4000 spectrometer (Ocean Optics, Inc, FL 34698, USA). The Spectrometer was controlled by the software SpectraSuite (SpectraSuite, Spectroscopy software, Ocean Optics Inc. 2008, FL 34698, USA).

Actiwatch Spectrum^TM^ devices were used to document the diurnal rhythm of the light change. Two devices documented the light change in one of the observation room areas and ten devices were mounted in the corridors in each of the ICUs. These were mounted vertically on the walls at a height of 1.5 meter and spaced to cover the areas. The vertical light measurements using Actiwatch are not comparable with the Hagner sensor measurements, since the spatial sensor response of Actiwatches is not cosine. Actiwatches do not measure the light from incidence angles of the light exceeding 60° (Figueiro et al. 2013; Price et al. 2012). Moreover, the spectral response of the Actiwatch white light output does not follow the photopic luminous efficiency function, V(λ) (Figueiro et al. 2013; Price et al. 2012). Thus, prior to the setup all Actiwatches were calibrated against a newly purchased reference lux sensor (Hanger® EC1-X, Instr. Nr. 54211 with connected Detector EC1-X, Instr. Nr. 54211, Sweden). Calibration was done at midday under overcast conditions where the reference sensor and all Actiwatches measured aligned and perpendicular to the direction of the sun. Synchronized Actiwatch white light output and photopic illuminance (reference lux) were compared and used to find calibration factors for each Actiwatch. The average calibration factor (reference lux/white light) for Actiwatch white light used was approx. 0.6. Calibration factors of the Actiwatch colour output, named by their associated colour perceptions as red (R light), green (G light) and blue (B light) were found by normalising to the average R, G and B light response, respectively. The three wavelength bands for Actiwatch R, G and B light have earlier been characterised (Figueiro et al. 2013; Price et al. 2012).

Figuerio MG, Hamner R, Bierman A, Rea MS (2013). Comparisons of three practical field devices used to measure personal light exposures and activity levels. Lighting Res Technol. 45:421-434.

Price LLA, Khazova M, Hagan JBO (2012). Performance assessment of commercial circadian personal exposure devices. Lighting Res Technol 44:17–26.
