# Supplementary material for: Shift work and quality of sleep: effect of working in designed dynamic light
Source: Int Arch Occup Environ Health. 2015 Apr 19;89:49–61. doi: 10.1007/s00420-015-1051-0 (PMC4700071; doi:10.1007/s00420-015-1051-0)
Supplement: Supplementary file 4 — Supplementary material 4 (DOC 24 kb) [file 420_2015_1051_MOESM4_ESM.doc]

**Statistical model for melatonin profiles (Figure 1)**

Data on log scale

name: <unnamed>

log: /Users/rholst/Dropbox/Projects/IRS/Vejle/Hanne Irene Jensen/Lysprojekt/Melatonin/FinalModel.smcl

log type: smcl

opened on: 2 Mar 2015, 15:42:25

. do "/var/folders/7j/xd5ckss96dz_k14yg4tv13jhy7nmnk/T//SD42013.000000"

. mi estimate,post: xtmixed mela_0_1 c.cos_hour##i.sted c.sin_hour##i.sted ||id:,mle

Multiple-imputation estimates Imputations = 50

Mixed-effects ML regression Number of obs = 713

Group variable: id Number of groups = 113

Obs per group: min = 2

avg = 6.3

max = 7

Average RVI = 0.0888

Largest FMI = 0.1411

DF adjustment: Large sample DF: min = 2484.33

avg = 6964.03

max = 11251.51

Model F test: Equal FMI F( 5,24654.9) = 43.48

Prob > F = 0.0000

---------------------------------------------------------------------------------

mela_0_1 | Coef. Std. Err. t P>|t| [95% Conf. Interval]

----------------+----------------------------------------------------------------

cos_hour | .1261848 .0118499 10.65 0.000 .1029554 .1494141

1.sted | .0128983 .0165893 0.78 0.437 -.019622 .0454186

|

sted#c.cos_hour |

1 | -.0208235 .0166274 -1.25 0.211 -.0534223 .0117752

|

sin_hour | .104876 .0112917 9.29 0.000 .0827339 .1270182

|

sted#c.sin_hour |

1 | -.0132432 .0158231 -0.84 0.403 -.0442674 .0177811

|

_cons | 1.125226 .011684 96.30 0.000 1.102323 1.148129

---------------------------------------------------------------------------------

------------------------------------------------------------------------------

Random-effects Parameters | Estimate Std. Err. [95% Conf. Interval]

-----------------------------+------------------------------------------------

id: Identity |

sd(_cons) | .0560417 .0056216 .046038 .0682192

-----------------------------+------------------------------------------------

sd(Residual) | .0945612 .0028219 .0891884 .1002577

------------------------------------------------------------------------------
